# Structure and activation mechanism of human sweet taste receptor

**DOI:** 10.1038/s41422-025-01156-x

**Published:** 2025-08-04

**Authors:** Haolan Wang, Xiao Chen, Yaxin Dai, Shabareesh Pidathala, Yiming Niu, Chen Zhao, Siyu Li, Liang Wang, Chia-Hsueh Lee

**Affiliations:** 1 Department of Structural Biology, St. Jude Children’s Research Hospital, Memphis, TN USA; 2 Laboratory of Chromosome and Cell Biology, The Rockefeller University, New York, NY USA; 3 Department of Biochemistry and Molecular Biology, University of Florida, Gainesville, FL USA

**Keywords:** Cryoelectron microscopy, Cell biology

Dear Editor,

Taste perception is initiated in the taste buds by cells that express G protein-coupled receptors (GPCRs) and ion channels tuned to specific modalities — sweet, bitter, umami, salty, and sour.^1^ Sugars and other sweet-tasting compounds are detected by the sweet taste receptor, a member of the class C GPCR family.^2,3^ The sweet receptor system evolved to prioritize energy-dense nutrients, engaging central reward pathways that reinforce sugar cravings and promote excessive calorie intake, contributing to obesity, type 2 diabetes, and other metabolic diseases. Though generally considered safe, use of artificial sweeteners has been associated with altered appetite regulation and increased cardiometabolic risk; moreover, non- or low-caloric sweeteners, such as sucralose, aspartame, and advantame, activate the sweet receptor but may not fully replicate the downstream effects of natural sugars (Supplementary information, Note S1). Therefore, elucidating the molecular basis of sweet receptor signaling and understanding how natural and artificial sweeteners engage the receptor may inform the rational design of next-generation sweeteners.

The sweet receptor is a heterodimer composed of TAS1R2 and TAS1R3,^3,4^ two type 1 taste receptor family members that share the distinctive architecture of class C GPCRs: a large extracellular Venus flytrap (VFT) domain, responsible for ligand recognition; a cysteine-rich domain (CRD); and a seven-transmembrane (7TM) domain that mediates G protein coupling. The molecular mechanisms by which diverse ligands engage the sweet receptor to trigger activation and sweet sensation remain largely unresolved. To address this gap, here we present cryo-electron microscopy (cryo-EM) structures of the sweet receptor in the inactive, unliganded state, as well as in the activated, sucralose- and advantame-bound states.

We studied sweet receptors comprising human TAS1R2 and either human or mouse TAS1R3, which share 73% sequence identity. The human/mouse heterodimer was shown to respond to both natural sugars and artificial sweeteners.^5^ To validate the function of our sweet receptor constructs, we evaluated their responses to sweeteners by calcium imaging assays^6^ (Supplementary information, Fig. S1a, c). Consistent with previous studies, the human/mouse sweet receptor responded to sweeteners neotame and advantame (EC_50_: 3.7 μM and 1.7 μM, respectively) in a manner comparable to the human/human receptor (EC_50_: 2.7 μM and 0.9 μM, respectively). We purified both human/mouse and human/human receptors from mammalian cells, and the former showed a sharper elution profile on size-exclusion chromatography (Supplementary information, Fig. S1b, d), suggesting that it may be more biochemically homogeneous.

We first determined the structure of the human/mouse sweet receptor in the absence of sweeteners. Through 3D classification, we observed substantial intrinsic mobility of the receptor. The extracellular domains tilt toward or away from the membrane, with an angular range of up to 11° (Supplementary information, Figs. S2, S3, and Table S1). While the orientation between extracellular and transmembrane domains can vary, each domain remains nearly identical across individual classes. To improve the resolution, we combined all particles to generate a global reconstruction at 3.1 Å resolution, then performed local refinement focused on the VFT/CRD region or the 7TM domain, obtaining final resolutions of 2.9 Å and 3.6 Å, respectively (Supplementary information, Figs. S2, S4). In the unliganded (apo) state, TAS1R2 and TAS1R3 show a highly asymmetric architecture, and their extracellular domains are markedly tilted relative to the membrane plane (Fig. 1a; Supplementary information, Fig. S3). These features differ from other heteromeric class C GPCRs, such as GABA_B_ receptor and heteromeric mGluRs, which adopt an orientation perpendicular to the membrane plane.^7,8^

**Fig. 1:**
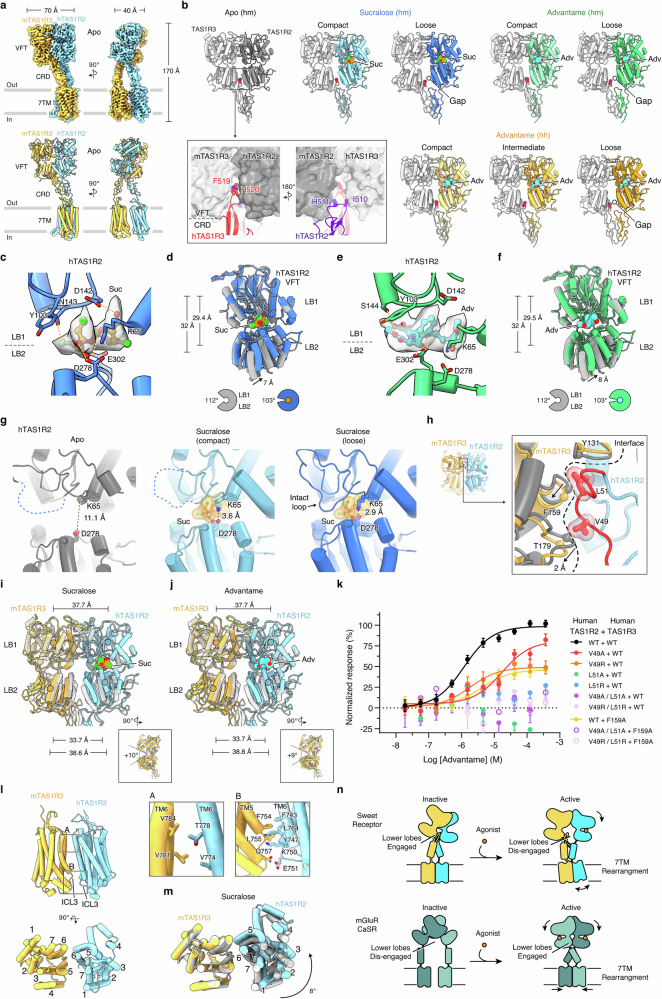
Ligand recognition and activation of the sweet taste receptor. **a** Cryo-EM density (top) and model (bottom) of the receptor in the apo state. **b** Conformations of the sweet receptor in the presence of sweeteners. Top row, human TAS1R2 and mouse TAS1R3 (hm) in the apo, sucralose- or advantame-bound state. Bottom row, human TAS1R2 and human TAS1R3 (hh) in the advantame-bound state; inset, interface between the inter-subunit VFT cleft and the CRD loops. **c** Sucralose-binding pocket in the TAS1R2 VFT domain, showing density of sucralose (Suc). Yellow dashed lines indicate polar interactions. **d** Clamshell closure induced by sucralose binding, illustrated by superimposing TAS1R2 VFT lobes in the apo (gray) and sucralose-bound loose (blue) states, aligned by LB1. Distances between the centers of mass (COMs) of LB1 and LB2: apo, 32 Å; sucralose-bound: 29.4 Å. Bottom, clamshell opening angles. **e** Advantame-binding pocket in the TAS1R2 VFT domain, showing density of advantame (Adv). Yellow dashed lines indicate polar interactions. **f** Clamshell closure induced by advantame binding, illustrated by superimposing the TAS1R2 VFT lobes in the apo (gray) and sucralose-bound loose (blue) states, aligned by LB1. Distances between COM of LB1 and LB2: apo, 32 Å; advantame-bound: 29.5 Å. Bottom, clamshell opening angles. **g** Conformational transitions of TAS1R2 VFT from apo to the sucralose-bound states. Disordered regions are shown as dashed lines. **h** Sweetener-induced rearrangement of the TAS1R2/TAS1R3 VFT interface in the sucralose-bound loose state. The loop from TAS1R2 is shown in red; TAS1R2 and TAS1R3 are colored blue and yellow, respectively. Inset, superposition of compact (gray) and loose (color) states showing the inter-subunit interface. Cα of selected residues in TAS1R3 shown as spheres. **i**, **j** Separation of TAS1R2 and TAS1R3 VFT domains induced by sucralose (**i**) or advantame (**j**). TAS1R2 and TAS1R3 in the apo state are colored light gray, and in the sweetener-bound (loose) state in blue and yellow, respectively. The COM of each individual lobe is shown as a filled circle; the distance between the LB1s, shown at the top, remains unchanged. The distances between the LB2s: apo, 33.7 Å; sweetener-bound: ~39 Å. Insets, change in the clamshell opening angle of TAS1R3 VFT. **k** Responses of human TAS1R2/TAS1R3 mutants measured by cell-based Ca²⁺ assays. Data are shown as mean ± SEM; wild type (WT), *n* = 29; mutants, *n* = 3–15. EC_50_: WT, 1.2 μM; TAS1R2 V49A, 17.1 μM; TAS1R2 V49R, 1.8 μM; TAS1R3 F159A, 4.0 μM. **l** 7TM domain interfaces of the sweet receptor in the apo state. Insets, residues lining the major interfaces between 7TM of TAS1R2 and TAS1R3. **m** Receptor activation triggers remodeling of the 7TM interface. TAS1R2 and TAS1R3 are colored in the sucralose-bound (loose) states, or gray in the apo state. **n** A schematic diagram illustrating the unique activation mechanism of the sweet receptor.

In our apo sweet receptor structure, the two subunits pack closely against each other at both the upper and lower lobes of the VFT domains, as well as at the 7TM domains (Fig. 1a, b). This sharply contrasts with the apo states of mGluRs and the calcium-sensing receptor (CaSR), where the dimer interface is restricted to the VFT upper lobes (LB1s), while the lower lobes (LB2s) and 7TM domains remain largely disengaged.^9,10^ The GABA_B_ receptor shows additional interactions between the 7TM domains, but their VFT LB2s remain separated.^7^ The tight packing of the sweet receptor is facilitated by interactions between VFT domains and CRDs, with the VFT LB2s held together by loops extending from the CRDs (Fig. 1b, inset; Supplementary information, Fig. S5a). Phe519 and His520 from TAS1R3 CRD are wedged deeply into the cleft between the two LB2s, effectively clasping them together, whereas equivalent residues Ile510 and His511 from TAS1R2 also extend into the VFT layer. This configuration reinforces the dimer interface and would enable the VFT domain of one subunit to directly communicate with the CRD of the other subunit, potentially allowing efficient cross-subunit allosteric coupling. Notably, such an arrangement differs from other class C GPCRs, where the VFT layer and the CRD remain structurally separated.^9,10^ We tested the functional relevance of this atypical VFT–CRD interface by mutating human TAS1R3 Phe514 and His515 (equivalent to mouse Phe519 and His520) and TAS1R2 Ile510 and His511, to either Ala or Glu (found at the corresponding positions in CaSR). Receptor activity was moderately altered by TAS1R3 F514A/H515A or TAS1R2 I510A/H511A mutations, but substantially impaired when the Ala mutations were combined or when the residues were mutated to Glu (Supplementary information, Fig. S5b).

These unique structural features indicate that the sweet receptor adopts a more rigid inactive conformation in the apo state, compared to other class C GPCRs, and follows a distinct activation mechanism. To investigate this mechanism, we determined the structures of the sweet receptor in the presence of sweeteners sucralose and advantame (Fig. 1b; Supplementary information, Figs. S6–S8 and Table S1). The two compounds, approved for use in human foods, represent distinct chemotypes: sucralose is a chlorinated analog of sucrose, and advantame is an ultra-potent sweetener derived from aspartame. In the presence of sweeteners, the human/mouse sweet receptor adopts two distinct conformations, which we refer to as compact and loose (Fig. 1b). In the compact conformation, the VFT LB2s remain packed; in the loose conformation, LB2s of the two subunits disengage. We also determined the structures of the human/human receptor in the presence of advantame; in addition to the compact and loose conformations, we observed an intermediate state with partial separation between the LB2s (Fig. 1b; Supplementary information, Fig. S8). Notably, the loose conformation accounted for 30%–50% of the particles across all datasets, suggesting that it represents a relevant state. This ligand-induced conformational change in the sweet receptor fundamentally differs from that of other class C GPCRs, where agonist binding brings the LB2s together.^8–11^

In the presence of sucralose, we observed a prominent ligand density in the TAS1R2 VFT domain (Fig. 1c; Supplementary information, Fig. S4b), consistent with previous functional studies showing that sucralose binds exclusively to TAS1R2.^3,12^ The VFT LB1 and LB2 form a clamshell-like structure, and sucralose adopts a U-shape that fits snugly within the cleft between the lobes. Its chlorinated fructose and glucose rings are oriented nearly side by side, in a vertical arrangement, allowing multiple polar interactions with residues lining the interlobe pocket: the chlorine interacts with Asn143; the hydroxyl and chlorine groups participate in a H-bonding network with polar residues including Asp142, Asp278, and Glu302. Additionally, sucralose’s pose is stabilized by hydrophobic interactions with Tyr103. Compared to the apo state, sucralose binding induces closure of the TAS1R2 VFT clamshell, with the bottom of LB2 lifting by up to 7 Å (Fig. 1d), reducing the clamshell opening from 112° (apo) to 105° (compact) and further to 103° (loose) (Fig. 1d; Supplementary information, Fig. S9a). Thus, the loose state shows the highest degree of TAS1R2 VFT closure, whereas the compact state may correspond to an intermediate along the activation trajectory. Because sucralose is an analog of natural sugars, its binding mode likely reflects how natural sweet compounds engage the receptor.

Advantame also yields well-defined ligand density in the TAS1R2 VFT pocket of both human/mouse and human/human receptors (Fig. 1e; Supplementary information, Fig. S4c, d). Advantame adopts a bent, arched pose within the clamshell cleft, with its Phe/Asp dipeptide core and vanillyl aromatic ring extending across the clamshell. Advantame engages TAS1R2 Ser144 through polar contacts, and its hydroxyl and amine groups form hydrogen bonds with conserved Asp142, Asp278, and Glu302. Although its interactions with TAS1R2 differ from those of sucralose, advantame elicits a comparable conformational change: the bottom of LB2 lifts by up to 8 Å, and the clamshell opening decreases from 112° (apo) to 104° (compact) and 103° (loose) (Fig. 1f; Supplementary information, Fig. S10a). Advantame is derived from aspartame and shares the dipeptide-based moiety; thus, our observations may recapitulate key interactions with aspartame, a widely used artificial sweetener, and explain sweet receptor activation by amino acid-based sweeteners.

The closure of the TAS1R2 VFT clamshell induced by sweetener binding leads to the formation of salt bridges between LB1 Lys65 and LB2 Asp278, which are brought into proximity in the compact state and engage further to form stable interactions in the loose state (Fig. 1g). In addition, a loop in TAS1R2 VFT LB1 (around residues 45–57), which is disordered in the apo and compact states, becomes ordered in the loose state (Fig. 1g) and inserts into the interface between TAS1R2 and TAS1R3. It interacts with the region surrounding Tyr131 and Phe159 of TAS1R3, pushing against an adjacent loop near Thr179, which is displaced by ~2 Å (Fig. 1h). These TAS1R3 residues are conserved across human and mouse, and this local displacement propagates across the TAS1R3 VFT, ultimately shifting the relative positioning of TAS1R2 and TAS1R3. As a result, the TAS1R3 VFT LB2 disengages from TAS1R2, and the distance between the two LB2s increases by ~5 Å (Fig. 1i, j), producing the loose state. By contrast, in the compact state, the inter-lobe interface remains largely preserved relative to the apo state, with the distance between the LB2s changing by only 0.7 Å (Supplementary information, Figs. S9b, S10b). We also observed an opening in the TAS1R3 VFT clamshell, with its opening increasing by 9°–10° in the loose state compared to the apo conformation (Fig. 1i, j, insets). This structural asymmetry of the VFT domains upon ligand binding underscores the non-equivalent functional roles of TAS1R2 and TAS1R3.

To our knowledge, this rearrangement and insertion of TAS1R2 LB1 loop during activation have not been observed in other class C GPCRs. To investigate its functional relevance, we mutated Val49 and Leu51 of TAS1R2 to either Ala or Arg (the equivalent residues in TAS1R3) and measured the human/human receptor activity (Fig. 1k). The mutations impaired sweetener-induced signaling, especially when Leu51 was mutated. Since TAS1R2 Leu51 interacts with TAS1R3 Phe159, we also mutated Phe159 and observed a similarly weakened response to the sweetener. These data highlight the importance of the interaction between the TAS1R2 VFT loop and TAS1R3 for sweet receptor activation.

Our observations suggest two key events during the activation of the sweet receptor. First, agonist binding induces clamshell closure in TAS1R2, accompanied by a stabilizing salt bridge that defines the compact state. Second, the TAS1R2 loop inserts into the inter-subunit interface, displacing TAS1R3 and triggering the separation of the VFT lower lobes, thereby forming the loose state. This mechanism is observed with both sucralose and advantame (Fig. 1j; Supplementary information, Fig. S10c–e) and recapitulated in the human/human receptor bound to advantame (Supplementary information, Fig. S11), indicating a universal feature of sweet receptor activation.

How do sweeteners promote activation of the 7TM domain? In the apo state, the 7TM domains of TAS1R2 and TAS1R3 interact through two interfaces (Fig. 1l), a configuration that is markedly different from that seen in other apo-state class C GPCRs (Supplementary information, Fig. S12a). Sucralose binding causes rearrangement of the inter-TM7 interfaces (Fig. 1m), likely altering the contacts observed in the apo state. We discuss how sweeteners promote activation of the 7TM domains, as well as the relevance of the mobility of the extracellular domains, in Supplementary information, Note S2.

Cryo-EM structures of the sweet taste receptor in complex with sweeteners aspartame and sucralose have been recently reported.^13,14^ The sweetener-bound structures in those studies closely resemble our compact state (Supplementary information, Fig. S12b), whereas the conformation of the 7TM domains (in the absence of G proteins) is similar to that observed in our apo structure. Importantly, those studies did not reveal the loose conformation that we describe here, a state that appears critical for receptor activation. These discrepancies may arise from differences in expression systems or construct design. Nonetheless, their structural and mutagenesis findings, along with prior functional studies,^12,15^ support the ligand-binding residues identified in our structures.

In closing, our work offers important new insights and a mechanistic framework for how sugars and artificial sweeteners trigger the sweet sensation. By capturing both apo and sweetener-bound states, we are able to infer the conformational changes that underlie receptor activation. Unlike other class C GPCRs, such as mGluR, CaSR and GABA_B_ receptors, where agonists stabilize inter-subunit VFT interactions to promote activation (Supplementary information, Fig. S12c), the sweet receptor is activated by the rearrangement of a TAS1R2 VFT loop, which destabilizes interactions between the lower VFT lobes and the VFT–CRD interfaces (Fig. 1n; Supplementary information, Fig. S12c). It is tempting to hypothesize that sweeteners that prolong the loose conformation of the receptor may elicit a stronger perception of sweetness. This structural understanding may guide the development of sweet receptor modulators with potential applications in dietary regulation.

## Supplementary information

Supplementary information

## Data Availability

Cryo-EM maps and coordinates have been deposited in the EMDB and wwPDB with following accession numbers. Apo: EMD-70726 (global); EMD-70727 and PDB 9OPW (VFT/CRD); EMD-70728 (VFT); EMD-70729 and PDB 9OPX (7TM); EMD-70730 and PDB 9OPY (class 3). Sucralose-bound: EMD-70731 and PDB 9OPZ (VFT, compact); EMD-70732 and PDB 9OQ0 (7TM, compact); EMD-70733 and PDB 9OQ1 (VFT, loose); EMD-70734 (7TM, loose). Advantame-bound: EMD-70735 and PDB 9OQ2 (VFT, compact); EMD-70736 (7TM, compact); EMD-70737 and PDB 9OQ3 (VFT, loose); EMD-70738 (7TM, loose). Human/human advantame-bound: EMD-70739 and PDB 9OQ4 (VFT, compact); EMD-70740 and PDB 9OQ5 (VFT, intermediate); EMD-70741 and PDB 9OQ6 (VFT, loose).
