# Supplementary material for: Structure and activation mechanism of human sweet taste receptor
Source: Cell Res. 2025 Aug 4;35(10):775–8. doi: 10.1038/s41422-025-01156-x (PMC12484785; doi:10.1038/s41422-025-01156-x)
Supplement: Supplementary file 1 — Supplementary information [file 41422_2025_1156_MOESM1_ESM.pdf]

Supplementary information

Structure and activation mechanism of human sweet taste receptor

Haolan Wang[1*], Xiao Chen[1*], Yaxin Dai[1], Shabareesh Pidathala[1], Yiming Niu[2], Chen Zhao[3], Siyu Li[1], Liang Wang[1], and Chia-Hsueh Lee[1]

[1]Department of Structural Biology, St. Jude Children's Research Hospital, Memphis, TN, USA.

[2]Laboratory of Chromosome and Cell Biology, The Rockefeller University, New York, NY, USA.

[3]Department of Biochemistry and Molecular Biology, University of Florida, Gainesville, FL, USA.

*These authors contributed equally: Haolan Wang, Xiao Chen

Correspondence: chiahsueh.lee@stjude.org

Supplementary Notes

S1: additional references on sugar and sweeteners

The sweet receptor system evolved to prioritize energy-dense nutrients, engaging central reward pathways that reinforce sugar cravings and promote excessive calorie intake[16,17], contributing to obesity, type 2 diabetes, and other metabolic diseases[18–20]. Though generally considered safe, use of artificial sweeteners has been associated with altered appetite regulation and increased cardiometabolic risk[21–28]; moreover, non- or low-caloric sweeteners such as sucralose, aspartame, and advantame activate the sweet receptor but may not fully replicate the downstream effects of natural sugars[23,29–31].

S2: additional discussion

Upon sucralose binding, in the loose state the 7TM domains of TAS1R2 undergo a 8° counterclockwise rotation, as viewed from the extracellular side (Fig. 1m). A similar rotation is also observed in the compact state. These conformational changes lead to a rearrangement of the two inter-subunit interfaces, likely altering or weakening the contacts observed in the apo state. However, the limited local resolution does not allow confident identification of the specific interactions that are altered. We also note that such rotation was not observed in the advantame-bound structures, where the 7TM domains are arranged similarly to the apo state. The local resolution of the 7TM domains in the advantame-bound structures is only 4.2–4.5 Å (Supplementary information, Fig. S7), suggesting greater flexibility compared to the sucralose-bound structures. This likely reflects conformational heterogeneity that is difficult to resolve with the current data, and further classification using a larger dataset may help uncover conformational states similar to the one identified with sucralose. Alternatively, under the experimental conditions used, advantame may trap the 7TM domains in a pre-active state. It is also possible that different sweeteners induce varying degrees of 7TM rotation. Future studies will be necessary to determine how this reorganization facilitates G protein coupling and signal transduction, and how the presence or absence of G proteins influences the conformational states of the receptors, both of which are key to fully understanding the activation mechanism.

In the apo state, the sweet receptor is tilted, and the extracellular domain is mobile. The relevance of this arrangement remains to be seen, but it may influence the ability of the VFT to fluctuate between conformations. In the ligand-bound and activated states, the receptor remains tilted; however, we did not observe distinct classes with varying tilt angles.

Materials and Methods

Cell-based calcium imaging assay

Expi293 cells stably expressing $G\alpha_{15}$-$gust_{44}$ were grown and maintained in high glucose Dulbecco's modified Eagle medium (Thermo Fisher Scientific, 11965092) supplemented with 10% fetal bovine serum (Thermo Fisher Scientific, A5670801) and 1× penicillin-streptomycin-glutamine (Thermo Fisher Scientific, 10378016) in a 37 °C incubator with 5% CO_2. At 70% confluency, the cells were transiently transfected using FuGENE 4K with encoding TAS1R2, TAS1R3 (as described in the next section, without the mVenus tag), and Ric-8A[32], at a 1:1:1 ratio. The cells were seeded into poly-D-lysine (Thermo Fisher Scientific, A3890401) coated 96-well black wall clear bottom plate (Corning, 3904) at a density of 50,000 cells/well and cultured in low-glucose Dulbecco's modified Eagle medium (Thermo Fisher Scientific, 10567014) supplemented with 1% dialyzed fetal bovine serum (Thermo Fisher Scientific, 26400044), 16 h after transfection.

Cells were assayed 48 h after transfection. After washing with assay buffer (Hanks' balanced salt solution supplemented with 20 mM HEPES pH7.5), cells were incubated at 37 °C in the dark with 100 µL of assay buffer containing 4 µM Cal-520 AM (AAT Bioquest, 21131) and 0.04% Pluronic F-127 (AAT Bioquest, 20053). After 1.5 h, the dye-containing buffer was removed, and cells were incubated with 100 µL fresh assay buffer for 15 min at 37 °C in the dark. This buffer was then replaced with another 100 µL of fresh assay buffer, and the assay plate was incubated in FlexStation 3 Multi-Mode Microplate Reader (Molecular Devices) at 37 °C for 20 min before the reading started.

Fluorescent signals were measured every 1.54 s for a period of 80 s, with an excitation wavelength of 485 nm and an emission wavelength of 525 nm, with a cutoff at 515 nm. An initial baseline read of 20 s was performed, followed by injection of 50 µL of 3× compound solution at a rate of 16 µL/s.

The baseline (F_0) was calculated by averaging raw fluorescence signals from 1 s to 20 s. The response to stimulus ($\Delta F/ F_0$) was determined by calculating the ratio of the maximum raw fluorescence signal between 28 and 51 seconds minus the baseline (ΔF), to the baseline fluorescence (F_0). The responses were baseline corrected using the no-stimulus control. The EC_{50} was determined using the three parameters nonlinear regression algorithm in GraphPad.

Expression and purification of the sweet receptor

The complementary DNA encoding human TAS1R2, mouse TAS1R3, or human TAS1R3 was cloned into a modified pEG BacMam vector[33]. The TAS1R2 construct consisted of an N-terminal H7 signal peptide[34], a Twin-Strep tag, and a GGGGS linker, followed by residues 22–839 of human TAS1R2. Three mutations (I132S, S212N, and D231G) were incorporated to enhance expression[35]. The TAS1R3 constructs consisted of an N-terminal H7 signal peptide, an ALFA tag, and a GGGGS linker, followed by residues 21–858 of mouse TAS1R3 or residues 21–853 of human TAS1R3, and included a C-terminal 3C protease cleavage site and an mVenus tag. Expression cassettes containing individual genes were amplified and assembled into the pBIG1a vector using the biGBac method[36]. Multigene constructs containing human TAS1R2/mouse TAS1R3 or human TAS1R2/human TAS1R3 were used for large-scale protein expression.

The sweet receptor was expressed in Expi293F cells (Gibco, A14527) using the BacMam system. Baculoviruses were generated by transfecting Sf9 cells (ATCC, CRL-1711) with bacmids using the Mirus TransIT-Insect reagent (Mirus Bio, MIR 6100). After one or two rounds of amplification, the viruses were used for cell transduction. When suspension cell cultures reached a density of 4×10^6 cells per mL after growth at 37 °C, baculoviruses (10% (v/v)) were added to initiate transduction. After 16–18 h, the cultures were supplemented with 10 mM sodium butyrate, and the temperature was shifted to 25 °C. Cells were harvested 60 h after transduction and stored at −80 °C.

Frozen cell pellets were thawed at room temperature and resuspended in a hypotonic buffer (10 mM Tris pH 8.0, 5 mM NaCl, 5 mM KCl, 2 mM $MgCl_2$, protease inhibitor cocktail, benzonase, and 2 mM NaATP) for 30 min on ice. The cell lysate was centrifuged at 39,800 × g for 30 min to sediment crude membranes. The resulting membrane pellet was mechanically homogenized in TBS buffer (20 mM Tris pH 8.0, 500 mM NaCl, and protease inhibitor cocktail). The suspension was solubilized with 1% (w/v) lauryl maltose neopentyl glycol (LMNG) and 0.2% (w/v) cholesteryl hemisuccinate (CHS) for 90 min at 4 °C. The solubilized material was centrifuged at 39,800 × g for 45 min, and the supernatant was incubated with ALFA Selector CE resin for 2 h at 4 °C. Resin was then washed with 40 column volumes of wash buffer A (40 mM Tris pH 8.0, 300 mM NaCl, 150 mM KCl, 4 mM $MgCl_2$, 4 mM NaATP, 0.005% GDN, 0.0005% CHS), followed by 10 column volumes of wash buffer B (20 mM Tris pH 8.0, 300 mM NaCl, 0.005% GDN, 0.0005% CHS). Protein was eluted with wash buffer B supplemented with 0.8 mg/mL ALFA elution peptide, concentrated, and further purified by gel-filtration chromatography on a Superose 6 Increase column equilibrated with buffer C (20 mM Tris pH 8.0, 300 mM NaCl, 0.001% GDN, 0.0001% CHS). Peak fractions containing protein were pooled and concentrated to ~8.0 mg/mL. For the sucralose-bound and advantame-bound complexes, ligands were added to all purification buffers at final concentrations of 5 mM and 1 mM, respectively.

Prior to cryo-EM grid preparation, the samples were further supplemented with either 35 mM sucralose or 4 mM advantame.

Cryo-EM sample preparation and data acquisition

For all cryo-EM experiments, a 3.5-µL volume of sample was applied to plasma-cleaned UltrAuFoil R1.2/1.3 300-mesh grids (Quantifoil) under 100% humidity at 10 °C. The grids were blotted for 3.0 s, plunge-frozen in liquid ethane using a Vitrobot Mark IV (Thermo Fisher Scientific), and loaded onto a 300 kV Titan Krios G3 microscope equipped with an energy filter. Images were collected using EPU software. Raw movie stacks were recorded on a K3 camera at a physical pixel size of 0.649 Å, with a nominal defocus range of 1.1–2.1 µm. Each micrograph was exposed for ~1.7 s, fractionated into 60 frames, with a dose rate of 0.99–1.03 e^- per $Å^2$ per second. The data collection parameters are summarized in Table S1.

Cryo-EM data processing

The image stacks were gain-normalized and corrected for beam-induced motion using MotionCor2[37]. Defocus parameters were estimated from motion-corrected images using cryoSPARC4. Micrographs not suitable for further analysis were removed by manual inspections. Particle picking (using blob and template pickers) and 2D classifications were performed in cryoSPARC4[38]. After 2D classification, selected particles were used to train the Topaz particle-picking model[39]. Particles picked by Topaz were subjected to additional 2D classifications. Selected particles were combined with those from the blob and template pickers, and duplicates were removed. Iterative 3D classifications, including ab initio reconstructions and heterogeneous refinements, were then performed to remove suboptimal particles. Particles from selected classes were first refined using non-uniform refinement[40], followed by local refinements with soft masks covering the region of interest to further improve map quality. Mask-corrected FSC curves were calculated in cryoSPARC4, and reported resolutions are based on the 0.143 criterion.

Model building and refinement

Initial models of the human TAS1R2, mouse TAS1R3 and human TAS1R3 subunit were generated by AlphaFold[41]. These models were docked into the density maps using Chimera[42]. The model was then refined iteratively using Coot[43], ISOLDE[44], and

Phenix[45]. Structural model validation was performed using Phenix and MolProbity[46] (Table S1). Figures were prepared using PyMOL, Chimera, and ChimeraX[47].

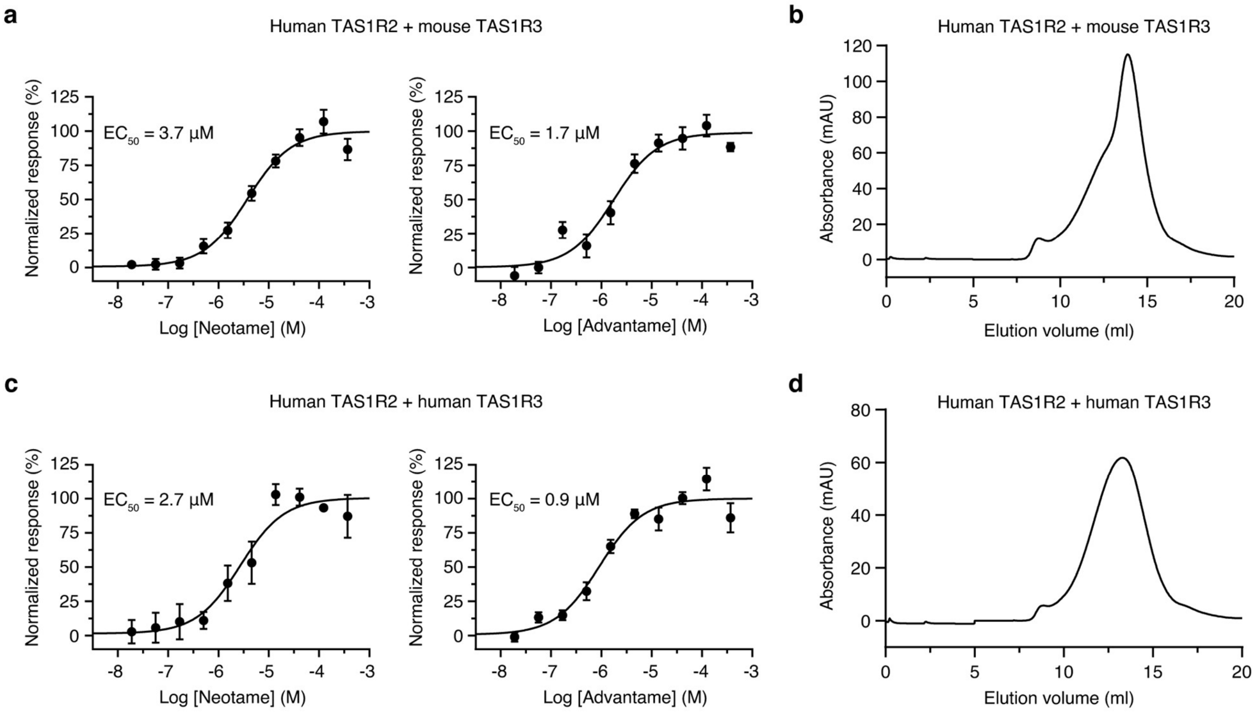

Fig. S1: Functional and biochemical characterizations of the sweet receptor.

a Sweetener-induced activation of human TAS1R2 and mouse TAS1R3 sweet receptor measured by the Ca^{2+} assay. Data are shown as mean ± SEM; n =4–8.

b Representative size exclusion chromatography profile of the human TAS1R2 and mouse TAS1R3 sweet receptor.

c Sweetener-induced activation of human TAS1R2 and TAS1R3 sweet receptor measured by the Ca^{2+} assay. Data are shown as mean ± SEM; n =4–8.

d Representative size exclusion chromatography profile of the human TAS1R2 and TAS1R3 sweet receptor.

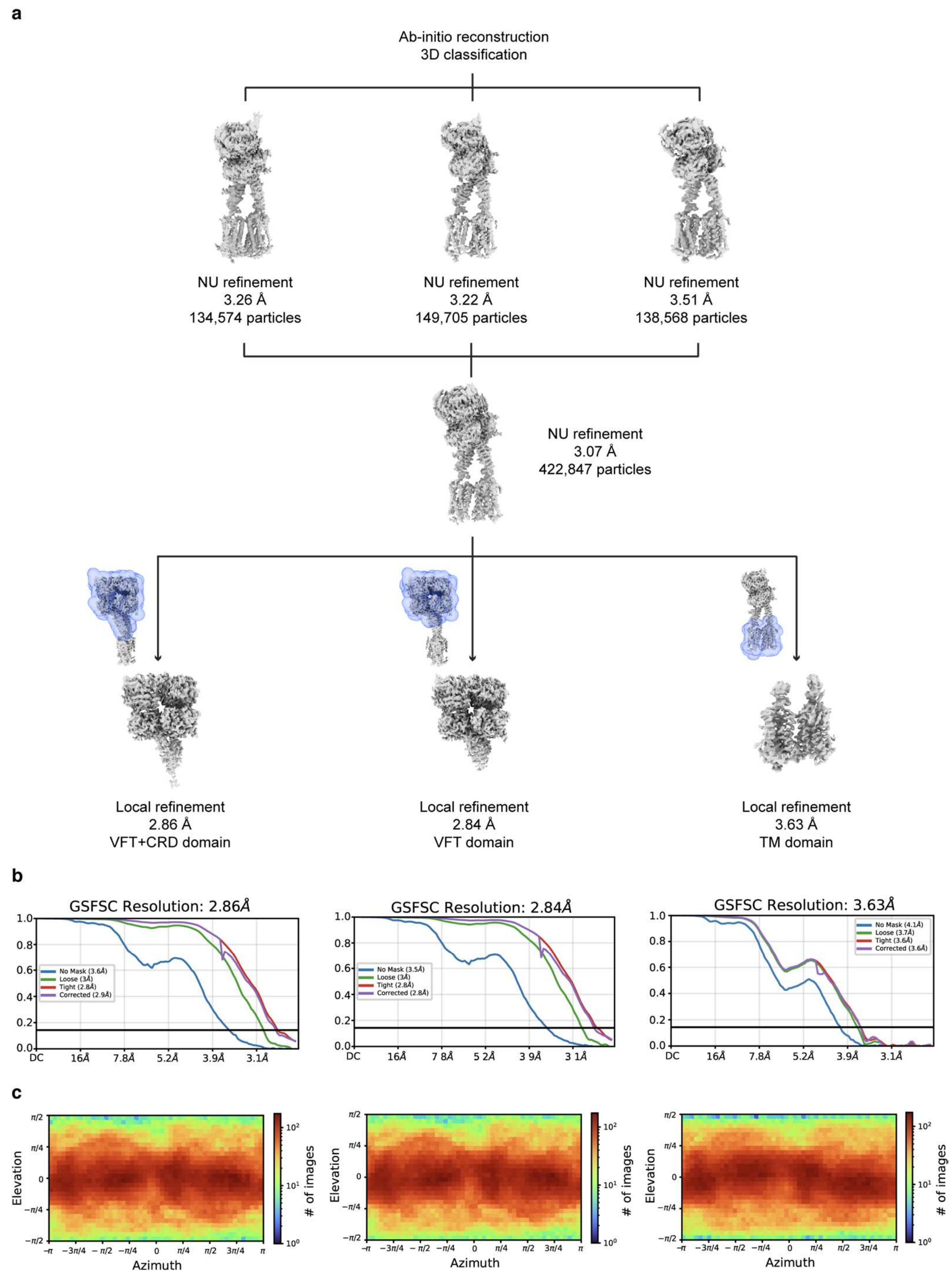

Fig. S2: Cryo-EM analysis of the sweet receptor in the apo state.

a Summary of image processing procedures for the apo state dataset (human/mouse). All processing steps were performed using cryoSPARC.

b Fourier shell correlation (FSC) curves between the two half-maps.

c Angular distribution of particles used in the final 3D reconstruction.

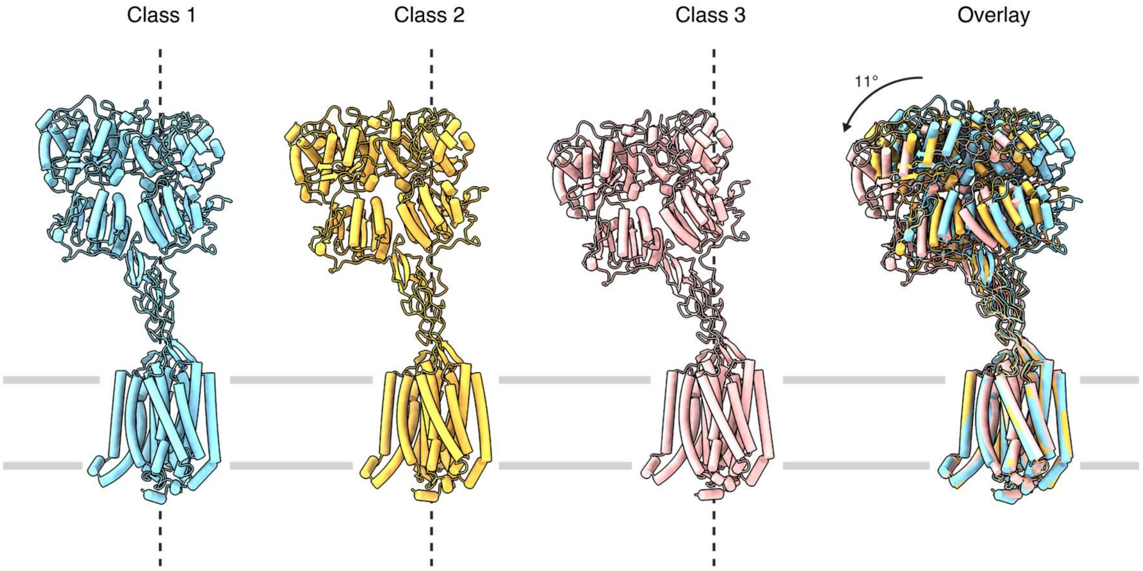

Fig. S3: Conformational variation of the sweet receptor in the apo state.

The sweet receptor (human/mouse) exhibits pronounced mobility and asymmetry. The extracellular domain is deviated from the central axis (dashed line) of the receptor. The overlay was generated by superimposing the structures on the 7TM domain.

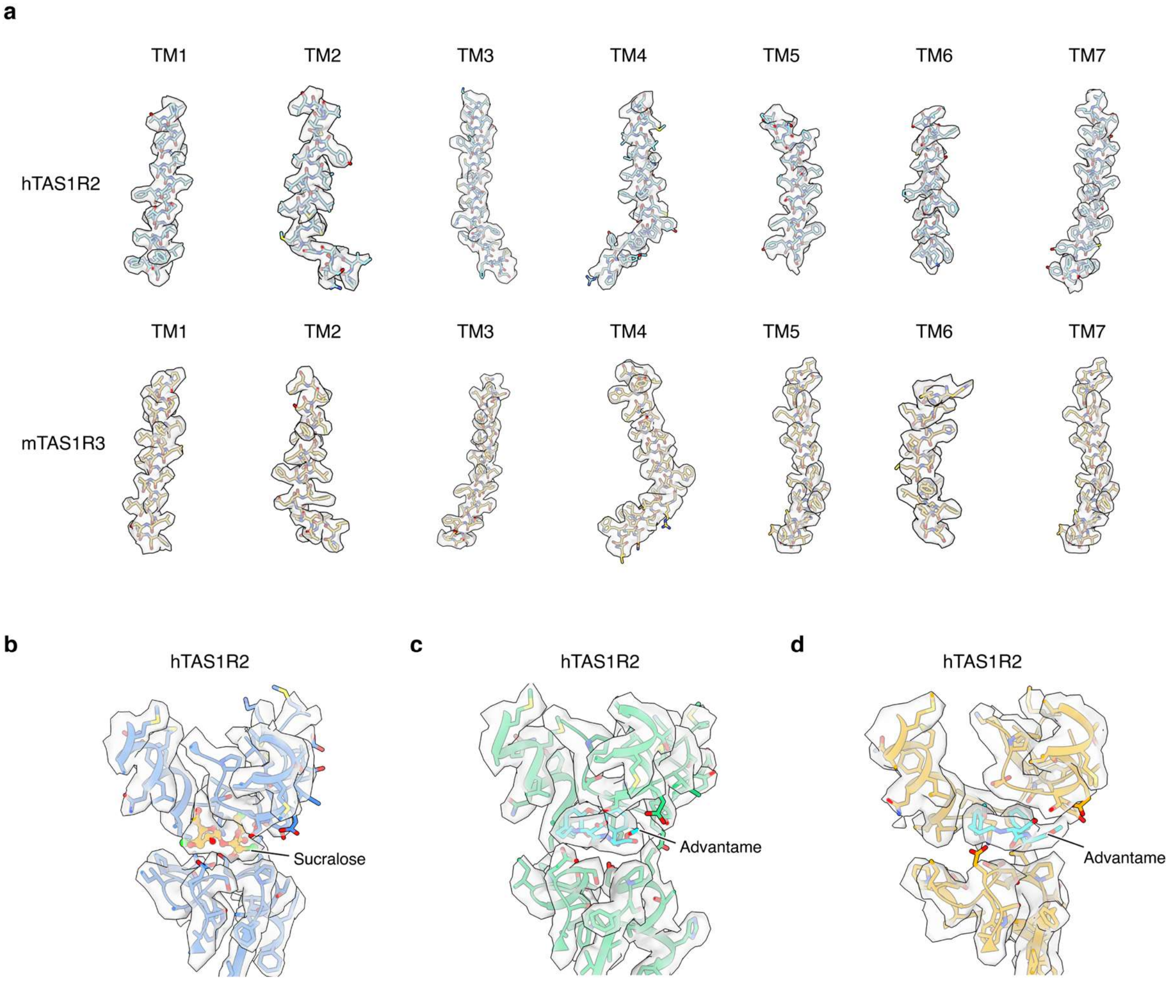

Fig. S4: Representative cryo-EM densities of sweet receptor structures.

a Cryo-EM densities of the transmembrane helices (human/mouse dataset) in the apo state.

b Cryo-EM densities of TAS1R2 bound to sucralose (human/mouse dataset), loose state.

c Cryo-EM densities of TAS1R2 bound to advantame (human/mouse dataset), loose state.

d Cryo-EM densities of TAS1R2 bound to advantame (human/human dataset), loose state.

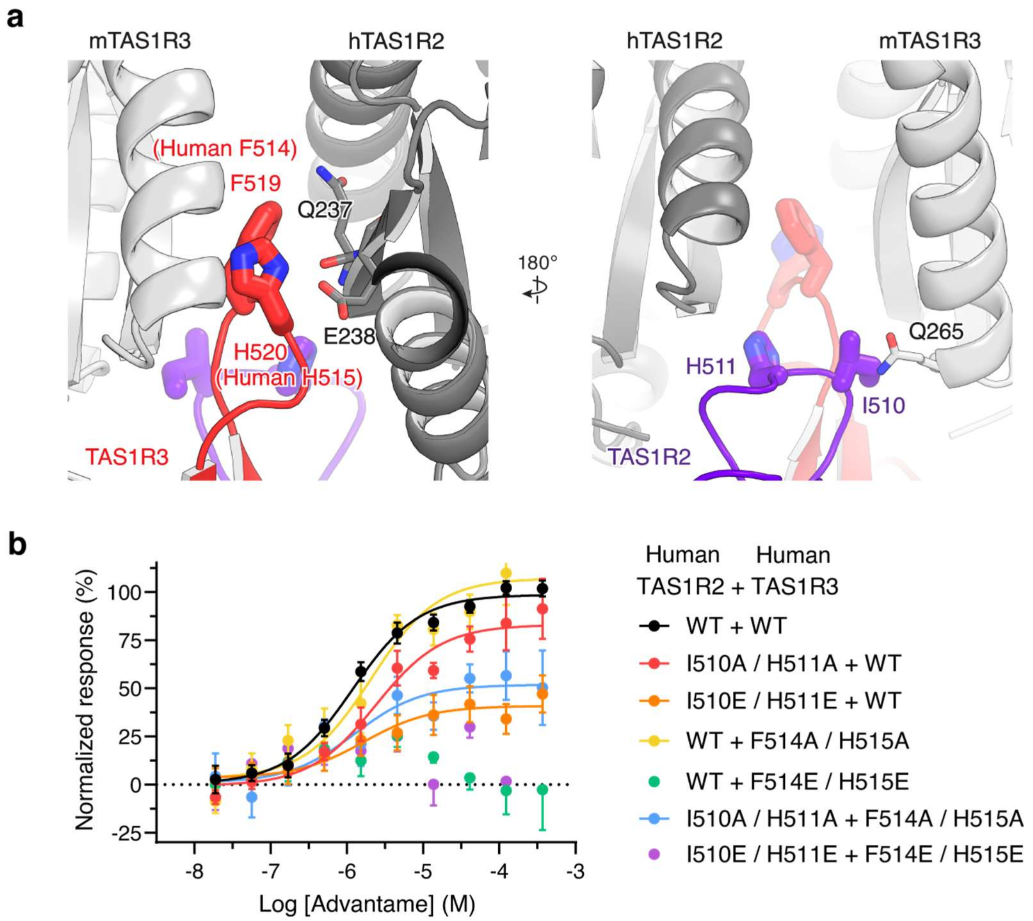

Fig. S5: Atypical VFT/CRD interface in the sweet receptor.

a Interactions between VFT and CRD in the apo state of the human/mouse receptor.

b Responses of human TAS1R2/human TAS1R3 receptor mutants measured by cell-based Ca^{2+} assays. Data are shown as mean ± SEM; WT, n = 29; mutants, n = 3–12. EC_{50}: WT, 1.2 µM; TAS1R2 I510A / H511A, 2.4 µM. TAS1R2 I510E / H511E, 1.7 µM; TAS1R3 F514A / H515A, 2.1 µM. TAS1R2 I510A / H511A + TAS1R3 F514A / H515A, 1.3 µM.

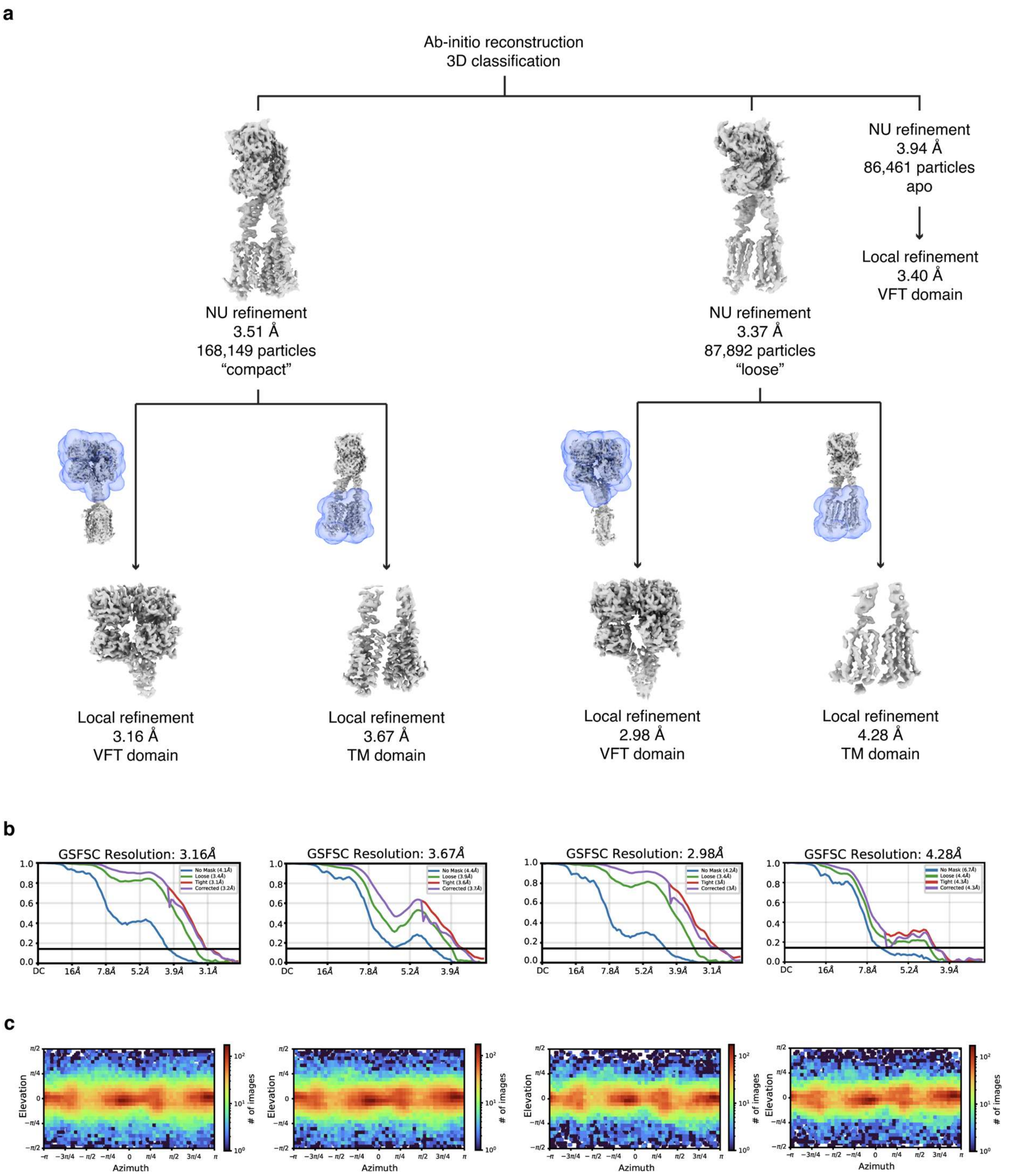

Fig. S6: Cryo-EM analysis of the sweet receptor in the sucralose-bound state.

a Summary of image processing procedures for the sucralose dataset (human/mouse). All processing steps were performed using cryoSPARC.

b Fourier shell correlation (FSC) curves between the two half-maps.

c Angular distribution of particles used in the final 3D reconstruction.

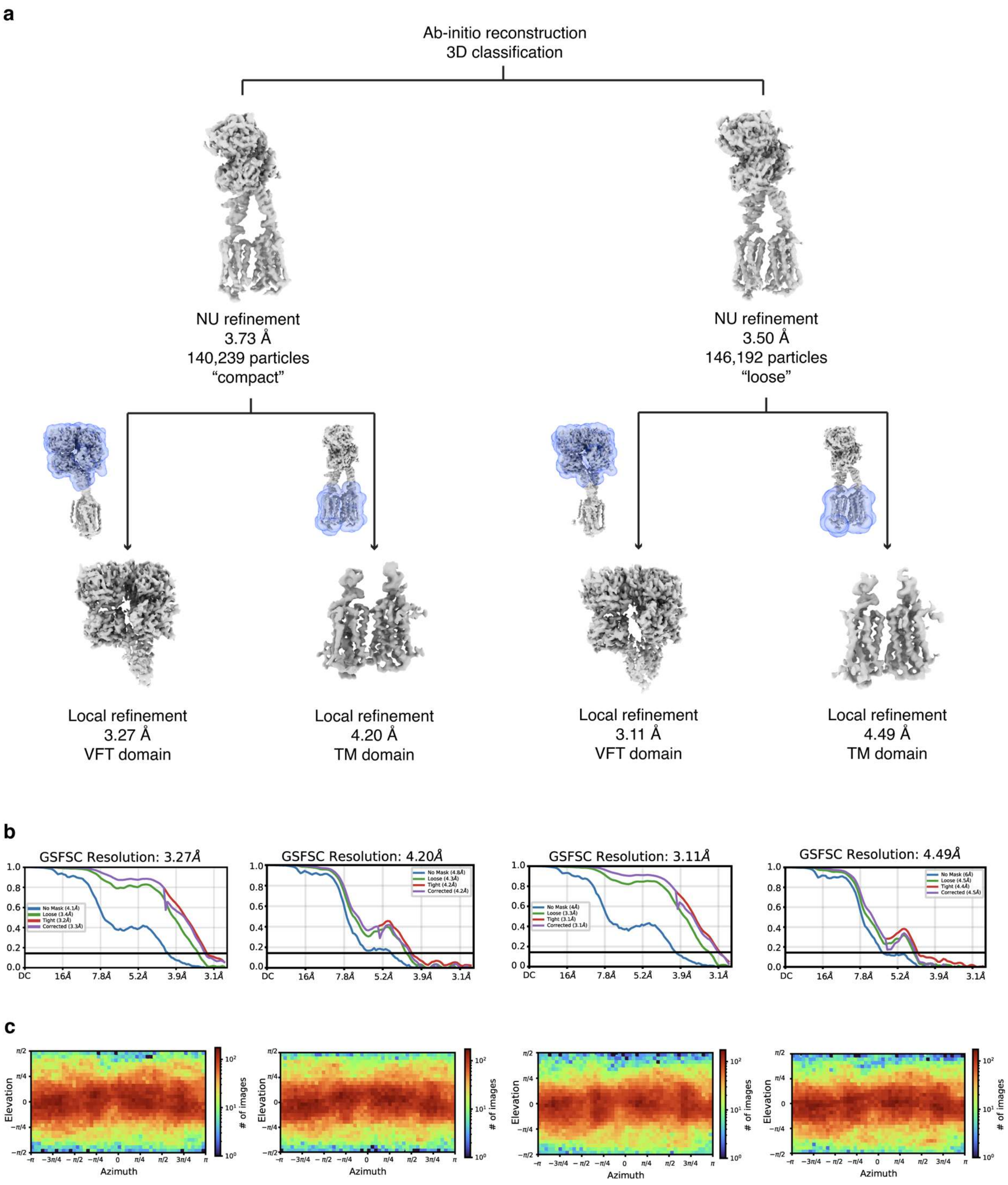

Fig. S7: Cryo-EM analysis of the sweet receptor in the advantame-bound state.

a Summary of image processing procedures for the advantame dataset (human/mouse). All processing steps were performed using cryoSPARC.

b Fourier shell correlation (FSC) curves between the two half-maps.

c Angular distribution of particles used in the final 3D reconstruction.

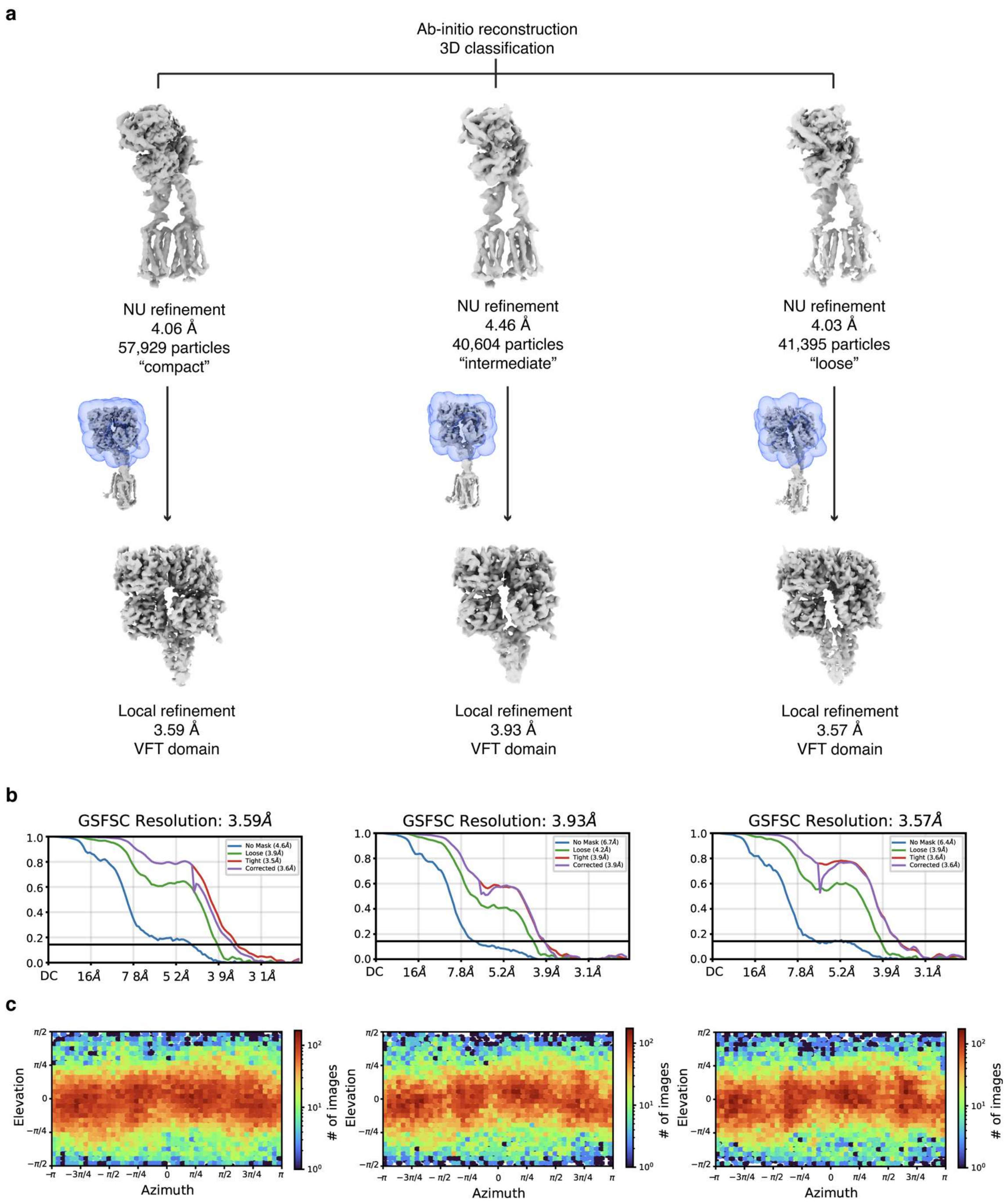

Fig. S8: Cryo-EM analysis of the sweet receptor (human/human) in the advantame-bound state.

a Summary of image processing procedures for the advantame dataset. All processing steps were performed using cryoSPARC.

b Fourier shell correlation (FSC) curves between the two half-maps.

c Angular distribution of particles used in the final 3D reconstruction.

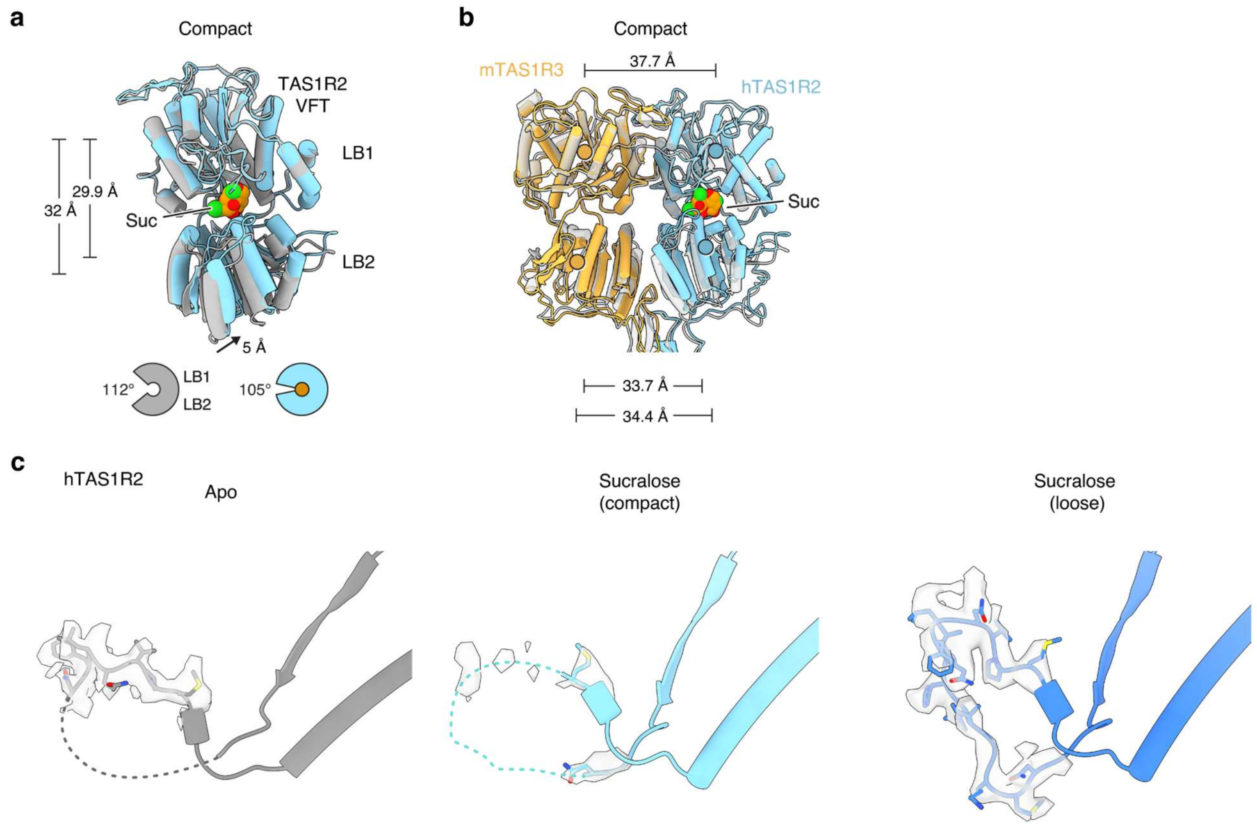

Fig. S9: Sucralose-induced conformational changes in the sweet receptor.

a Clamshell closure induced by sucralose, illustrated by superimposing the TAS1R2 VFT lobes in the apo (gray) and sucralose-bound compact (blue) states, aligned by LB1. The distances between COM of LB1 and LB2 lobes are indicated (Apo: 32 Å; sucralose: 29.9 Å). Clamshell opening angles are shown in the bottom schematics.

b Separation of TAS1R2 and TAS1R3 VFT domains induced by sucralose. TAS1R2 and TAS1R3 in the apo state are colored light gray. The subunits in the sweetener-bound (compact) state are shown in blue and yellow, respectively. The COM of individual lobes are shown as filled circles. The distance between the LB1 lobes is shown at the top and remains unchanged upon sucralose binding; thus, only one measurement is shown. The distances between the LB2 lobes are shown at the bottom (apo: 33.7 Å; sucralose-bound: 34.4 Å).

c Rearrangement and stabilization of the TAS1R2 loop from the apo state to the sucralose-bound state. Cryo-EM densities of this region are shown.

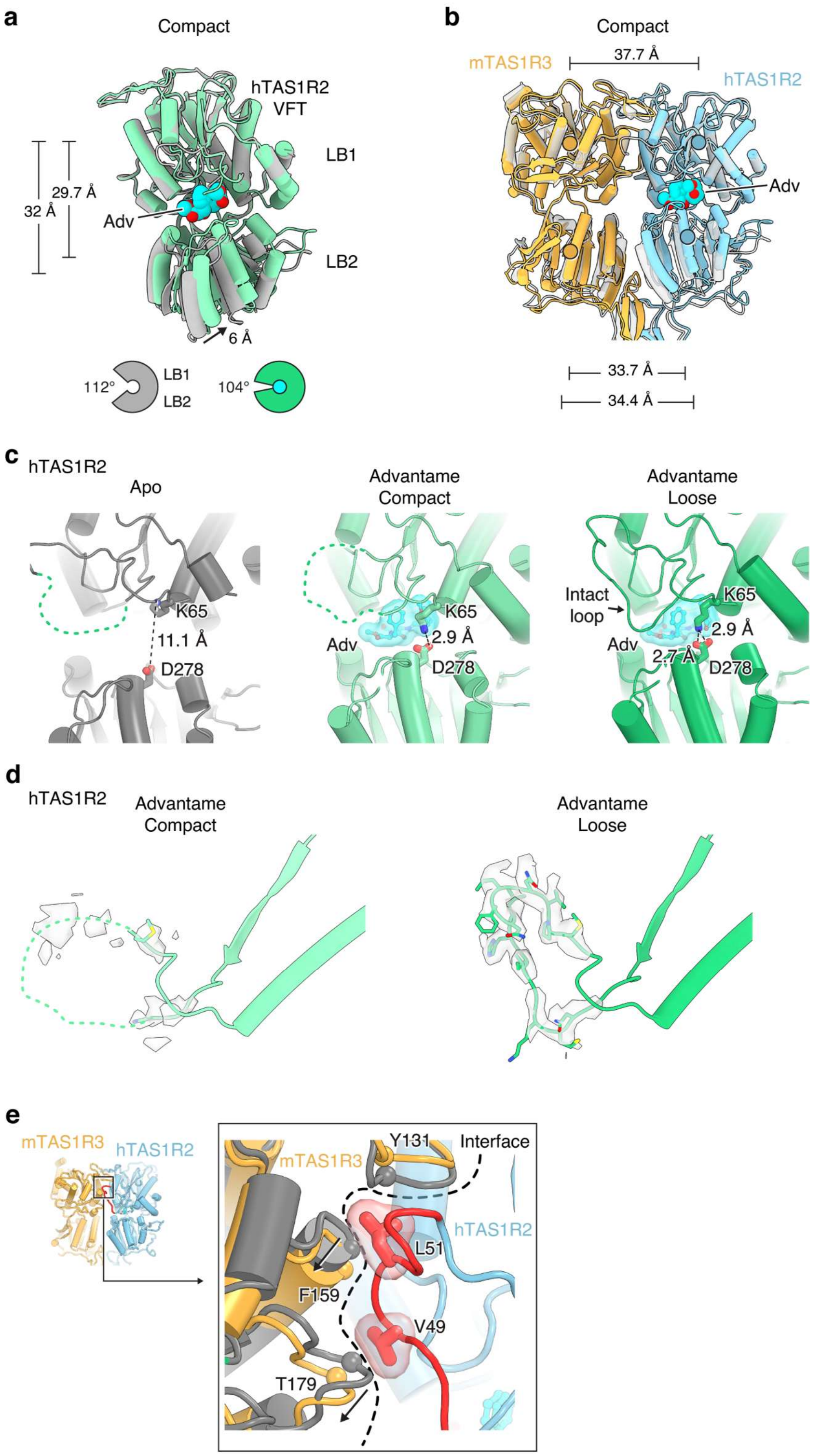

Fig. S10: Advantame-induced conformational changes in the sweet receptor.

a Clamshell closure induced by advantame, illustrated by superimposing the TAS1R2 VFT lobes in the apo (gray) and advantame-bound compact (green) states, aligned by LB1. The

distances between COM of LB1 and LB2 lobes are indicated (Apo: 32 Å; advantame: 29.7 Å). Clamshell opening angles are shown in the bottom schematics.

b Separation of TAS1R2 and TAS1R3 VFT domains induced by advantame. TAS1R2 and TAS1R3 in the apo state are colored light gray. The subunits in the sweetener-bound (compact) state are shown in blue and yellow, respectively. The COM of individual lobes are shown as filled circles. The distance between the LB1 lobes is shown at the top and remains unchanged upon advantame binding; thus, only one measurement is shown. The distances between the LB2 lobes are shown at the bottom (apo: 33.7 Å; advantame - bound: 34.4 Å).

c Conformational transitions of TAS1R2 VFT between the apo and two advantame-bound states. Disordered regions are shown as dash lines. Advantame is in surface and stick representation.

d Rearrangement and stabilization of the TAS1R2 loop (around residues 45–57) from the apo state to the advantame-bound state. Cryo-EM densities of this region are shown.

e Advantame-induced interactions between the VFT LB1 from TAS1R2 and TAS1R3 in the sucralose-bound loose state. The loop from TAS1R2 is shown in red; TAS1R2 and TAS1R3 are colored blue and yellow, respectively. Inset, superposition of compact (gray) and loose (color) states showing rearrangement of the inter-subunit interface. The Cα of selected residues in TAS1R3 are shown as spheres.

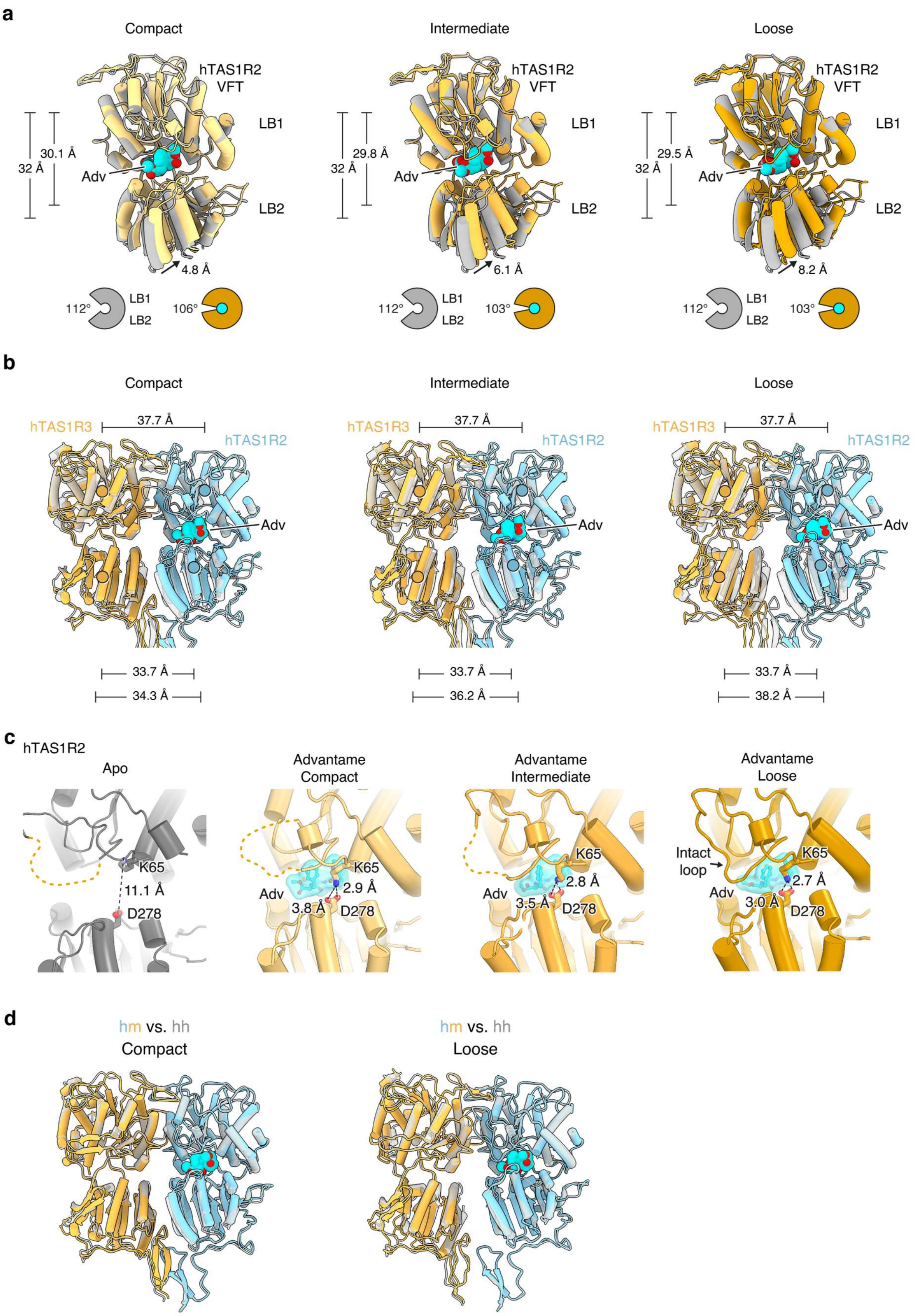
a
Compact
Intermediate
Loose
hTAS1R2
VFT
LB1
LB2
Adv
30.1 Å
32 Å
29.8 Å
29.5 Å
4.8 Å
6.1 Å
8.2 Å
112°
106°
103°
b
hTAS1R3
hTAS1R2
37.7 Å
33.7 Å
34.3 Å
36.2 Å
38.2 Å
c
hTAS1R2
Apo
Advantame
Compact
Advantame
Intermediate
Advantame
Loose
K65
11.1 Å
D278
2.9 Å
3.8 Å
2.8 Å
3.5 Å
2.7 Å
3.0 Å
Intact
loop
d
hm vs. hh
Compact
Loose

Fig. S11: Advantame-induced conformational changes in the human sweet receptor.

a Clamshell closure induced by advantame, illustrated by superimposing the TAS1R2 VFT lobes in the apo (gray) and advantame-bound compact (green) states, aligned by LB1. The distances between COM of LB1 and LB2 lobes are indicated. Clamshell opening angles are shown in the bottom schematics.

b Separation of TAS1R2 and TAS1R3 VFT domains induced by advantame. TAS1R2 and TAS1R3 in the apo state are colored light gray. The subunits in the advantame-bound state are shown in blue and yellow, respectively. The COM of individual lobes are shown as filled circles. The distance between the LB1 lobes is shown at the top and remains unchanged upon advantame binding; thus, only one measurement is shown. The distances between the LB2 lobes are shown at the bottom.

c Conformational transitions of TAS1R2 VFT from apo to the three advantame-bound states. Disordered regions are shown as dash lines. Advantame is in surface and stick representation.

d Comparison between the human/mouse and human/human receptor in the advantame-bound states. The structures from the two species are similar in both compact and loose states.

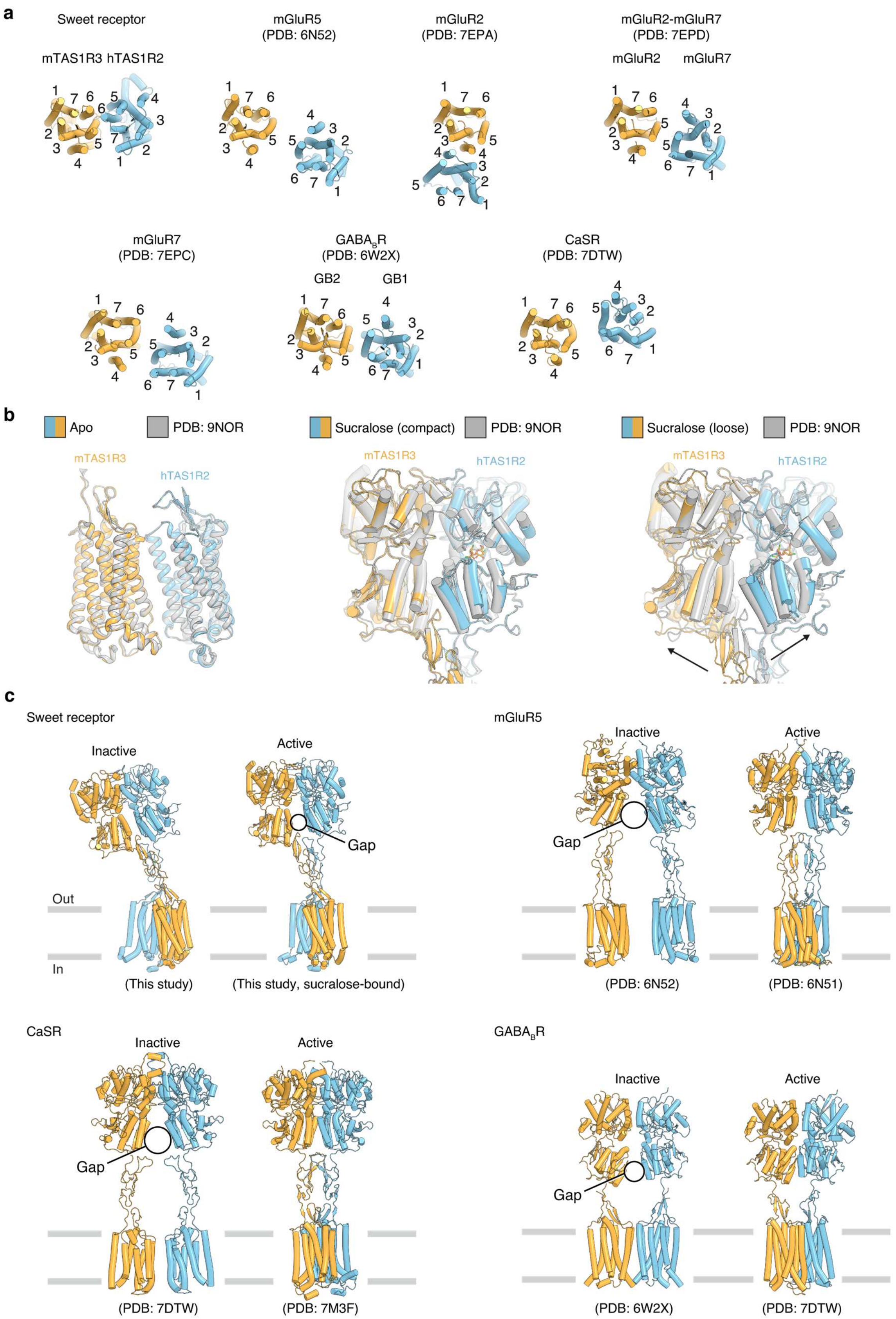

a
Sweet receptor
mTAS1R3 hTAS1R2
mGluR5
(PDB: 6N52)
mGluR2
(PDB: 7EPA)
mGluR2-mGluR7
(PDB: 7EPD)
mGluR2 mGluR7
mGluR7
(PDB: 7EPC)
GABA$_B$R
(PDB: 6W2X)
GB2 GB1
CaSR
(PDB: 7DTW)
b
Apo
PDB: 9NOR
Sucralose (compact)
PDB: 9NOR
Sucralose (loose)
PDB: 9NOR
mTAS1R3
hTAS1R2
c
Sweet receptor
Inactive
Active
Gap
Out
In
(This study)
(This study, sucralose-bound)
mGluR5
Inactive
Active
Gap
(PDB: 6N52)
(PDB: 6N51)
CaSR
Inactive
Active
Gap
(PDB: 7DTW)
(PDB: 7M3F)
GABA$_B$R
Inactive
Active
Gap
(PDB: 6W2X)
(PDB: 7DTW)

Fig. S12: Structural comparison of the sweet receptor.

a Arrangement of the 7TM domains of class C GPCRs in the apo state. The sweet receptor uses distinct interfaces to dimerize in the transmembrane region.

b Comparison between structures from this study and the previously reported sucralose-bound structure of the human sweet receptor. The reported structure (PDB: 9NOR) closely resembles the compact state observed in our study. In contrast, the loose state we identified is unprecedented and features a distinct inter-subunit arrangement.

c Structural comparison between inactive and active states of selected class C GPCRs.

Structure	Apo (global)	Apo (VFT/CRD)	Apo (VFT)	Apo (7TM)	Apo (class 3)
PDB		9OPW		9OPX	9OPY
EMDB	70726	70727	70728	70729	70730
**Data collection/ processing**					
Magnification	130,000x	130,000x	130,000x	130,000x	130,000x
Voltage (kV)	300	300	300	300	300
Pixel size (Å)	0.649	0.649	0.649	0.649	0.649
Defocus range (μm)	1.1~2.1	1.1~2.1	1.1~2.1	1.1~2.1	1.1~2.1
Electron exposure ($e^{-}/Å^{2}$)	60.6	60.6	60.6	60.6	60.6
Symmetry imposed	C1	C1	C1	C1	C1
Initial particles (No.)	~12 millions	~12 millions	~12 millions	~12 millions	~12 millions
Final particles (No.)	422,847	422,847	422,847	422,847	138,568
Map resolution (Å)	3.07	2.86	2.84	3.63	3.51
FSC threshold	0.143	0.143	0.143	0.143	0.143
Map resolution range (Å)	42.3-2.9	47.2-2.8	46.8-2.8	15.3-3.0	42.7-2.9
**Refinement**					
Model Resolution (Å)	NA	3.0	NA	3.7	3.6
FSC threshold	NA	0.5	NA	0.5	0.5
Map sharpening B-factor ($Å^{2}$)	-119.0	-111.6	-112.8	-140.9	-118.2
Model composition					
Non-hydrogen atoms		8106		4327	12388
Protein residues		1021		553	1570
Ligand		0		0	0
B-factors ($Å^{2}$)					
Protein		55.56		55.51	86.45
Ligand		NA		NA	NA
R.m.s. deviations					
Bond lengths (Å)		0.003		0.003	0.003
Bond angles (°)		0.574		0.581	0.621
Validation					
MolProbity score		0.77		0.75	1.01
Clashscore		0.81		0.80	1.58
Rotamers outliers (%)		0.00		0.00	0.00
Ramachandran plot (%)					
Favored		97.91		98.72	97.49
Allowed		2.09		1.28	2.51
Outliers		0.00		0.00	0.00

Table S1: Cryo-EM data collection, refinement and validation statistics

Structure	Sucralose (VFT, compact)	Sucralose (7TM, compact)	Sucralose (VFT, loose)	Sucralose (7TM, loose)	Advantame (VFT, compact)
PDB	9OPZ	9OQ0	9OQ1		9OQ2
EMDB	70731	70732	70733	70734	70735
**Data collection/ processing**					
Magnification	130,000x	130,000x	130,000x	130,000x	130,000x
Voltage (kV)	300	300	300	300	300
Pixel size (Å)	0.649	0.649	0.649	0.649	0.649
Defocus range (μm)	1.1–2.1	1.1–2.1	1.1–2.1	1.1–2.1	1.1–2.1
Electron exposure (e^-/Å^2)	61.6	61.6	61.6	61.6	60.4
Symmetry imposed	C1	C1	C1	C1	C1
Initial particles (No.)	~33 millions	~33 millions	~33 millions	~33 millions	~11 millions
Final particles (No.)	168,149	168,365	87,750	87,892	140,239
Map resolution (Å)	3.16	3.67	2.98	4.28	3.27
FSC threshold	0.143	0.143	0.143	0.143	0.143
Map resolution range (Å)	47.4–2.9	55.5–3.6	48.2–2.9	63.7–3.6	27.6–3.2
**Refinement**					
Model Resolution (Å)	3.3	4.0	3.4	NA	3.4
FSC threshold	0.5	0.5	0.5	NA	0.5
Map sharpening B-factor (Å^2)	-110.6	-133.8	-95.0	-177.1	-112.5
Model composition					
Non-hydrogen atoms	8147	4329	8251		8157
Protein residues	1024	553	1037		1024
Ligand	1	0	1		1
B-factors (Å^2)					
Protein	70.31	73.12	113.34		63.02
Ligand	62.09	NA	82.36		30.87
R.m.s. deviations					
Bond lengths (Å)	0.003	0.004	0.003		0.002
Bond angles (°)	0.634	0.740	0.649		0.578
Validation					
MolProbity score	1.36	1.17	1.21		0.72
Clashscore	3.74	2.28	2.58		0.69
Rotamers outliers (%)	0.00	0.00	0.00		0.00
Ramachandran plot (%)					
Favored	96.84	97.09	97.08		98.02
Allowed	3.16	2.91	2.92		1.98
Outliers	0.00	0.00	0.00		0.00

Structure	Advantame (7TM, compact)	Advantame (VFT, loose)	Advantame (7TM, loose)	Human/human Advantame (VFT, compact)	Human/human Advantame (VFT, intermediate)
PDB		9OQ3		9OQ4	9OQ5
EMDB	70736	70737	70738	70739	70740
**Data collection/ processing**					
Magnification	130,000x	130,000x	130,000x	130,000x	130,000x
Voltage (kV)	300	300	300	300	300
Pixel size (Å)	0.649	0.649	0.649	0.649	0.649
Defocus range (μm)	1.1–2.1	1.1–2.1	1.1–2.1	1.1–2.1	1.1–2.1
Electron exposure ($e^-/Å^2$)	60.4	60.4	60.4	59.3	59.3
Symmetry imposed	C1	C1	C1	C1	C1
Initial particles (No.)	~11 millions	~11 millions	~11 millions	~4.8 millions	~4.8 millions
Final particles (No.)	140,239	146,192	146,192	57,901	40,562
Map resolution (Å)	4.20	3.11	4.49	3.59	3.93
FSC threshold	0.143	0.143	0.143	0.143	0.143
Map resolution range (Å)	11.8–3.6	11.9–3.1	22.6–3.7	61.9–2.8	61.9–3.4
**Refinement**					
Model Resolution (Å)	NA	3.2	NA	3.9	3.3
FSC threshold	NA	0.5	NA	0.5	0.5
Map sharpening B-factor ($Å^2$)	-191.5	-106.2	-205.8	-118.5	-124.1
Model composition					
Non-hydrogen atoms		8261		8066	8151
Protein residues		1037		1015	1025
Ligand		1		1	1
B-factors ($Å^2$)					
Protein		63.98		114.07	86.56
Ligand		23.61		115.57	77.23
R.m.s. deviations					
Bond lengths (Å)		0.003		0.002	0.004
Bond angles (°)		0.611		0.603	0.698
Validation					
MolProbity score		0.93		0.84	0.95
Clashscore		1.54		0.76	1.50
Rotamers outliers (%)		0.11		0.00	0.00
Ramachandran plot (%)					
Favored		97.86		97.51	97.73
Allowed		2.14		2.49	2.27
Outliers		0.00		0.00	0.00

Structure	Human/human Advantame (VFT, loose)
PDB	9OQ6
EMDB	70741
**Data collection/ processing**	
Magnification	130,000x
Voltage (kV)	300
Pixel size (Å)	0.649
Defocus range (μm)	1.1–2.1
Electron exposure (e^-/Å^2)	59.3
Symmetry imposed	C1
Initial particles (No.)	~4.8 millions
Final particles (No.)	41,355
Map resolution (Å)	3.57
FSC threshold	0.143
Map resolution range (Å)	61.8–3.3
**Refinement**	
Model Resolution (Å)	
FSC threshold	0.5
Map sharpening B-factor (Å^2)	-102.6
Model composition	
Non-hydrogen atoms	8170
Protein residues	1028
Ligand	1
B-factors (Å^2)	
Protein	119.15
Ligand	85.82
R.m.s. deviations	
Bond lengths (Å)	0.003
Bond angles (°)	0.613
Validation	
MolProbity score	0.94
Clashscore	1.18
Rotamers outliers (%)	0.00
Ramachandran plot (%)	
Favored	97.45
Allowed	2.55
Outliers	0.00

References

16. DiFeliceantonio, A. G. *et al.* Supra-Additive Effects of Combining Fat and Carbohydrate on Food Reward. *Cell Metab.* **28**, 33-44.e3 (2018).

17. Veldhuizen, M. G. *et al.* Integration of Sweet Taste and Metabolism Determines Carbohydrate Reward. *Curr. Biol.* **27**, 2476-2485.e6 (2017).

18. Malik, V. S., Pan, A., Willett, W. C. & Hu, F. B. Sugar-sweetened beverages and weight gain in children and adults: a systematic review and meta-analysis123. *Am. J. Clin. Nutr.* **98**, 1084–1102 (2013).

19. Malik, V. S., Popkin, B. M., Bray, G. A., Després, J.-P. & Hu, F. B. Sugar-Sweetened Beverages, Obesity, Type 2 Diabetes Mellitus, and Cardiovascular Disease Risk. *Circulation* **121**, 1356–1364 (2010).

20. Bray, G. A. & Popkin, B. M. Dietary Sugar and Body Weight: Have We Reached a Crisis in the Epidemic of Obesity and Diabetes?: Health Be Damned! Pour on the Sugar. *Diabetes Care* **37**, 950–956 (2014).

21. Azad, M. B. *et al.* Nonnutritive sweeteners and cardiometabolic health: a systematic review and meta-analysis of randomized controlled trials and prospective cohort studies. *CMAJ Can. Med. Assoc. J. J. Assoc. Medicale Can.* **189**, E929–E939 (2017).

22. Witkowski, M. *et al.* The artificial sweetener erythritol and cardiovascular event risk. *Nat. Med.* **29**, 710–718 (2023).

23. Chakravartti, S. P. *et al.* Non-caloric sweetener effects on brain appetite regulation in individuals across varying body weights. *Nat. Metab.* **7**, 574–585 (2025).

24. Yunker, A. G. *et al.* Obesity and Sex-Related Associations With Differential Effects of Sucralose vs Sucrose on Appetite and Reward Processing: A Randomized Crossover Trial. *JAMA Netw. Open* **4**, e2126313 (2021).

25. Steffen, B. T. *et al.* Long-term aspartame and saccharin intakes are related to greater volumes of visceral, intermuscular, and subcutaneous adipose tissue: the CARDIA study. *Int. J. Obes.* **47**, 939–947 (2023).

26. Suez, J. *et al.* Personalized microbiome-driven effects of non-nutritive sweeteners on human glucose tolerance. *Cell* **185**, 3307-3328.e19 (2022).

27. Suez, J. *et al.* Artificial sweeteners induce glucose intolerance by altering the gut microbiota. *Nature* **514**, 181–186 (2014).

28. Wu, W. *et al.* Sweetener aspartame aggravates atherosclerosis through insulin-triggered inflammation. *Cell Metab.* **37**, 1075-1088.e7 (2025).

29. Wu, W. *et al.* Sweetener aspartame aggravates atherosclerosis through insulin-triggered inflammation. *Cell Metab.* **0**, (2025).

30. Dalenberg, J. R. *et al.* Short-Term Consumption of Sucralose with, but Not without, Carbohydrate Impairs Neural and Metabolic Sensitivity to Sugar in Humans. *Cell Metab.* **31**, 493-502.e7 (2020).
31. Zani, F. *et al.* The dietary sweetener sucralose is a negative modulator of T cell-mediated responses. *Nature* **615**, 705–711 (2023).
32. Fenech, C. J. *et al.* Ric-8A, a Gα protein guanine nucleotide exchange factor potentiates taste receptor signaling. *Front. Cell. Neurosci.* **3**, (2009).
33. Goehring, A. *et al.* Screening and large-scale expression of membrane proteins in mammalian cells for structural studies. *Nat. Protoc.* **9**, 2574–2585 (2014).
34. Haryadi, R. *et al.* Optimization of Heavy Chain and Light Chain Signal Peptides for High Level Expression of Therapeutic Antibodies in CHO Cells. *PLOS ONE* **10**, e0116878 (2015).
35. Park, J. *et al.* Structural architecture of a dimeric class C GPCR based on co-trafficking of sweet taste receptor subunits. *J. Biol. Chem.* **294**, 4759–4774 (2019).
36. Weissmann, F. *et al.* biGBac enables rapid gene assembly for the expression of large multisubunit protein complexes. *Proc. Natl. Acad. Sci. U. S. A.* **113**, E2564–E2569 (2016).
37. Zheng, S. Q. *et al.* MotionCor2 - anisotropic correction of beam-induced motion for improved cryo-electron microscopy. *Nat. Methods* **14**, 331–332 (2017).
38. Punjani, A., Rubinstein, J. L., Fleet, D. J. & Brubaker, M. A. cryoSPARC: algorithms for rapid unsupervised cryo-EM structure determination. *Nat. Methods* **14**, 290–296 (2017).
39. Bepler, T. *et al.* Positive-unlabeled convolutional neural networks for particle picking in cryo-electron micrographs. *Nat. Methods* **16**, 1153–1160 (2019).
40. Punjani, A., Zhang, H. & Fleet, D. J. Non-uniform refinement: adaptive regularization improves single-particle cryo-EM reconstruction. *Nat. Methods* **17**, 1214–1221 (2020).
41. Jumper, J. *et al.* Highly accurate protein structure prediction with AlphaFold. *Nature* **596**, 583–589 (2021).
42. Pettersen, E. F. *et al.* UCSF Chimera--a visualization system for exploratory research and analysis. *J. Comput. Chem.* **25**, 1605–1612 (2004).
43. Emsley, P., Lohkamp, B., Scott, W. G. & Cowtan, K. Features and development of Coot. *Acta Crystallogr. D Biol. Crystallogr.* **66**, 486–501 (2010).
44. Croll, T. I. ISOLDE: a physically realistic environment for model building into low-resolution electron-density maps. *Acta Crystallogr. Sect. Struct. Biol.* **74**, 519–530 (2018).
45. Afonine, P. V. *et al.* Real-space refinement in PHENIX for cryo-EM and crystallography. *Acta Crystallogr. Sect. Struct. Biol.* **74**, 531–544 (2018).
46. Chen, V. B. *et al.* MolProbity: all-atom structure validation for macromolecular crystallography. *Acta Crystallogr. D Biol. Crystallogr.* **66**, 12–21 (2010).

47. Goddard, T. D. *et al.* UCSF ChimeraX: Meeting modern challenges in visualization and analysis. *Protein Sci. Publ. Protein Soc.* **27**, 14–25 (2018).
